# Active imaginative listening—a neuromusical critique

**DOI:** 10.3389/fnins.2014.00251

**Published:** 2014-08-22

**Authors:** David Rosenboom

**Affiliations:** The Herb Alpert School of Music, California Institute of the ArtsValencia, CA, USA

**Keywords:** biofeedback, brain-computer music interface, music neuroscience, neuromusic, propositional music, self-organizing musical forms

## Abstract

The parallel study of music in science and creative practice can be traced back to the ancients; and paralleling the emergence of music neuroscience, creative musical practitioners have employed neurobiological phenomena extensively in music composition and performance. Several examples from the author's work in this area, which began in the 1960s, are cited and briefly described. From this perspective, the author also explores questions pertinent to current agendas evident in music neuroscience and speculates on potentially potent future directions.

## What is the music we are studying?

What is music? I hope we never see a day when we believe we know the answer. For that day would close down music as a viable art form. Music is a vast open space, in which the range of practices extant among the human species that can be *called* music—not counting other possible forms of intelligence—is too broad to be experienced in a human lifetime. Music is a dynamically evolving, cultural ecosystem; and it is not possible to nail down definitive predictions in what is fundamentally a continuously creative, self-organizing, emergent space with a vast *adjacent possible* (Kaufman, 2000). Musical forms are emergent, and the ways in which we interact with them evolve over time. Music and the brain most likely co-evolve, as has been posited for the brain and language (Deacon, 1997). Indeed, a recent study suggests that brain mechanisms for auditory beat perception, and further, neural structures capable of simulating and predicting the timing of rhythms, have evolved uniquely in humans (Patel and Iverson, 2014). We might extrapolate that if we take music in its broadest possible meaning, still unexplored aspects of music's coevolution with the brain may unveil powerful new insights about the very nature of human beings.

What is the agenda for music neuroscience now? In a quick sampling of sources, (ex. Hodges, 1996; Avazini et al., 2003, 2005; Peretz and Zatorre, 2003; Bella et al., 2009; Overy et al., 2012), one can find a range of motivations. For some, the object is to learn more about the brain, and music provides a particularly rich stimulus domain with which to study it. For others, the goal is to learn more about the enigmatic forms of human behavior *called* music. Certainly, the agenda for music neuroscience is already rich and diverse. It also includes providing a rich stimulus set with which to characterize auditory responses in the brain and seeking to understand neural networks involved in music perception and production. Neuroscientists also study comparative aspects of music perception in animals, psychoacoustics, the role of memory in musical performance, brain plasticity in learning to sing or play instrumental music, development of music perception in infants, how musical training may enhance acquisition of language and cognitive skills, the nature of brain impairments in music perception and production, and the value of music therapy in clinical populations.

Musical artists, along with many other groups, are interested in how music neuroscience can inform and inspire creative practices. A particular subgroup has been making great strides in techniques for Brain Computer Music Interface (BCMI)—(for numerous examples, see: Miranda and Castet, 2014). Some develop compositional models informed by ideas from music neuroscience and/or apply neurological data to musical structures (ex. Minciacchi, 2003). Others relate composition to mental states correlated with EEG data (ex. Wu et al., 2010). Applications in performance are wide ranging (ex. Lusted and Knapp, 1988). A broader survey—even just from the author's personal experiences—would enumerate many examples of artistic creation and learning informed by music neuroscience—(see more examples cited later in this article). Often, these musical artists operate with extremely broad views about the range of human activities and experiences that can be regarded as musical. (For example, see Rosenboom, 2000a for a discussion about *propositional music*, in which composers may invent new definitions of music as part of their artistic practice.) I believe it is very important that music neuroscientists take care to avoid overly narrow presumptions about what music *is* when designing experimental paradigms and what Ian Cross has called “… an inclusive delineation of the domain of music for such research,” (Cross, 2003). This may help facilitate the best possible, productive and collaborative energies, accompanied by informative, interdisciplinary communication, among a wide range of artists and scientists exploring neuromusical pursuits.

If we were to search for intelligence in outer space while presuming only closed models of what we believe intelligence *can be*, we might well miss manifestations of intelligence, the forms of which we *cannot know in advance*. Similarly, if we study the neuroscience of music limited by a priori assumptions about what music *is*, we might not learn from forms of musical engagement that we aren't prepared to recognize—(see Rosenboom, 2003b for further discussion). Rather than beginning with implicit definitions of music, even though they may facilitate the design of replicable experiments, music neuroscience might benefit from beginning with and periodically returning to the first principle of surveying the full range of what musical practitioners *consider to be music*, particularly master musicians, from diverse cultures and from traditional practices to the most contemporary and experimental. Informed choices can then be made about what to study and how to design useful experimental paradigms. Master musicians are master listeners, fully alert to all aspects of what composer Luciano Berio refers to as “… the ongoing dialog between the ear and the mind” (Berio, 2006). For master creative listeners, who through intensive practice can become hyper-aware of how they parse sound and construct endogenous musical memory engrams, listening itself can be elevated to the level of composition. To be sure, constraints on the dynamics of acculturation can result in a convergence on particular styles becoming prominent in specific cultural contexts and times. This dynamic, concomitant conditioning is in itself worthy of studying. N. M. Weinberger points out risks associated with using “highly specified music stimuli,” and that “… music neuroscience risks conceptual and empirical isolation, with consequent fragmentary understanding, if it fails to learn lessons from and benefits from these two fields of inquiry, which themselves have been undergoing a degree of fruitful synthesis” (Weinberger, 2014). In the end, it may be best to assume no more explicit definition of music than that given by composer-philosopher John Cage simply as “organization of sound” (Cage, 1967). I suggest further that a fundamental form of musical intelligence might be described as *active imaginative listening* to what each listener chooses intentionally to regard as musical. Some examples of paradigmatic risks follow.

In Western classical music, dating from a brief period of about two and a half centuries during which composition and performance became radically specialized and rigidly separated, the forms of compositions were largely teleological. They presented thematic statements with intentionally composed goals for their development. Diatonic harmony was about starting somewhere (exposition), moving away (development), and returning from tension (dissonance) to resolution (consonance). Of course, neuromusical studies with Western classical forms can be worthwhile and illuminating. However, in my personal experiences over 40 years collaborating professionally with musical masters from many parts of the Globe, I have found that in some cultures, teleological musical forms make no sense. In these communities, music may be regarded as a flowing stream, possibly with cycles upon cycles in their structures, and with no true concept of beginning or ending—(for example, in some indigenous African and contemporary experimental music). The practice of these musical forms may involve individuals or groups joining the streams and cycles at some point in time and leaving at another, while the streams and cycles continue endlessly. In still others, music is not seen as being separate from the surrounding soundscapes of nature, inside which it resides—(examples include Inuit music and contemporary soundscape music). In some cultures, terms for music and art are not endemic in their languages—(for example, in some tribes of Papua, New Guinea). They are simply natural aspects of daily life, not separate, not needing labels. Throughout most of music history and across most of the globe, composition, improvisation, and performance are not distinguished from each other, as they are in Western classical music. In many cultures, the term “improvisation” is not to be found. Composition and improvisation are not considered to be different or requiring specialized terminology. Improvisation is, instead, presumed to be a natural component of music making.

Quite naturally, music neuroscience often attempts to elucidate the functions of musical harmony and the perception of consonance and dissonance, and many useful results have come from this. It should be noted, however, that commonly held concepts of consonance and dissonance are somewhat ethnocentric Western ideas heavily dependent upon the tuning and scale systems in use. Many cultures do not recognize or use these terms as we do and may classify the intervals of musical scales according to different models, particularly if their music is primarily linear and monophonic, i.e., not based on simultaneously interacting parts. While recent studies do suggest that the auditory systems of infants are sensitive to Western harmonic constructions, such as major vs. minor and consonance vs. dissonance (Virtala et al., 2013), the effects of culturally determined listening strategies on brain function have also been noted (ex. Neuhaus, 2003). Even within Western classical music, intervals of pitch that are considered consonant or dissonant have evolved over time (Tenney, 1988). Intervals considered dissonant in one era may be considered consonant in another; and as Bregman is careful to point out, their musical functions may not concur with their psychoacoustic definitions (Bregman, 1990). Jazz has radically altered these classifications, sometimes referring to “color tones” that would otherwise be labeled dissonant. Other cultures, for example Balinese, intentionally tune sets of instruments to produce shimmering beats with pitches that are very close but slightly apart from one another. In other contexts these might also be considered dissonant or simply “out of tune.” Additionally, what may be considered consonant can be affected by tuning systems. When intervals are tuned to *Just* (rational, whole-number) ratios, perceived consonance may extend to intervals considered to be dissonant in non-*Just* (irrational) scales, like the equal tempered scale of the modern piano. In some musical practices, tuning and harmonic relationships are determined partially as function of listening time. For example, a chord normally expected to resolve from a dissonance to a consonance in Western diatonic harmony, may loose its resolution imperative, if it is tuned with rational intervals and listened to as a drone for a very long time. Such a chord may come to be perceived as perfectly settled, not needing to *go* anywhere. A variety of composers have exploited this phenomenon, for example, minimalist progenitor, La Monte Young, and others who followed (Poter, 2000). Finally, even classical diatonicism eventually gave way to dynamic chromaticism, in which the diatonic tonal matrix was stretched, as if on a rubber sheet. The components of its voice leading were subjected to individual prolongations, and chords became smeared into vaguely classifiable, musical verbs, not the discrete objects that make models for syntactic computation convenient. Attempts to produce quantitative measures of harmonic functions must be sensitive to experience in perceiving and processing complex pitch ratios and tolerance ranges for tunings associated with quasi-harmonic (ex. equal tempered), non-harmonic (irrational), and sub-harmonic (non-linear) pitch relationships. All of these can become extremely interesting with listening experience and have been used in music composition. Sutherland et al. (2013) may be developing useful methods in this regard.

Recent directions in contemporary music are very diverse—(for good surveys see Gann, 1997; Nyman, 1999; Zorn, 2000–2012; Cope, 2001). Some employ probabilities to create stochastic musical environments with measured predictability and scales of complexity, order, and disorder. Others develop systems for social ordering among participants in a performance or games for improvisation. Some composers work closely with emotion, meaning, expression, and narrative form, while others strive to eliminate all these things and produce only naturally pure, almost Platonic, sonic constructions. Many work with interactive models instead of the usual, one-way communication from composer to listener. Many contemporary scoring techniques offer choices to performers in how they move through musical material and/or employ methods for indeterminacy. Progressive jazz musicians, experimental singer-songwriters, turntable-ists, beat-loop musicians, gradual process composers, deep listening sonic meditators, circuit benders, drum circle players, noise bands, and auditory threshold minimalists all produce music far outside the presumptions of teleological, classical forms; and large audiences attest to their popularity and efficacy.

Truly exploratory musical artists are often frustrated by the investigations of music neuroscience, because they don't seem to be relevant to *their* music or *how they hear*. It is difficult, particularly for Westerners, to imagine the profound ways in which cognitive models of music can vary. Indeed, *proposed cognitive models of music* can be considered components of compositional techniques (Rosenboom, 1987). Truly creative music makers may build entire models of proposed worlds—what I call *propositional music*—to become the bases for their musical practices (Rosenboom, 2000a). So far, all we can truly identify as *givens* about music are: (1) music usually deals with organized sound, and (2) music making is usually, not always, a shared activity. The true breadth of what music *can be* suggests expanding the range of what music neuroscience might investigate. In my opinion, music neuroscience must strive to include *all* music in its exploration of the *whole brain*. Acknowledging that considerable work has been done in some of these areas, here's a brief, still incomplete, list of questions that might suggest places to start:

- What is a musical “event” or “entity,” and what are the roles of attention, perception, acculturation, and cognition as determinants for how individuals and groups identify them?
- What are the general principles by which the auditory nervous system and primary processing areas of the brain identify low-level structural elements in musical forms?
- What are the mechanisms of higher-level musical feature extraction, with respect to formal musical structures; is this process hierarchical, and what is the role of structural context—degrees of variance, stochastic qualities, ranges, and distributions of parametric values, etc.—in this process?
- Do clear neural concomitants exist for temporal gestalt perception? (See: Tenney, 1992 for a discussion of temporal gestalt perception.)
- What are the principles for and neural concomitants of how we parse musical forms when pitch and harmonic structures are not the primary organizing parameters in musical forms?
- Can we track neural substrates for how various acoustical parameters might be weighted relative to each other in parsing musical forms and sonic scenes?
- How can we characterize neural substrates for various non-tempered tuning systems; do neural network plasticity effects result from extensive exposure to these systems, and what is the special role of rational proportions in the perceptual organization of music?
- What are the principles by which we learn to discriminate and compare aspects of complexity in sonic streams?
- Are parsing principles for music that is largely improvised different from those involving fixed forms?
- What are the neural underpinnings for affective reactions to degrees of musical variance and complexity and why these can be different for non-musicians, musicians, and super-musicians?
- What are principles of perceptual organization for musical forms that are cyclical and not based on linear structures for teleological development, or modular, in which pathways through the musical materials are indeterminate and decided spontaneously by performers?
- Can we find neural concomitants for possible origins of music as a form of gesture communication, and can these be tracked and mapped in musical forms today?
- How are neural network processing resources applied to production and perception of complex rhythmical structures, which may be hierarchically organized in small to very large groups; what are the roles of short and long-term memory in this process, and how are the necessary motor skills for production best learned?
- How do we study music that is highly conceptual, perhaps involving only acts of self-directed listening? (See Oliveros, 2005 for interesting ideas on *deep listening*.)
- Can we use music to better understand the perceptual organization and cognitive modeling of time?
- Should we start with cross-cultural comparative studies about cognitive models at work in ideas about what music *is* and *can be*?

## Brief historical notes on extended musical interface with the human nervous system

By now, we have traversed nearly 60 years of creative investigations in which composers and allied artists have made works of music, visual art, kinetic art, theater, dance, interactive installation, and telepresent performance employing direct monitoring of biological phenomena, such as electroencephalogram (EEG), electromyogram (EMG), electrocardiogram (EKG), galvanic skin response (GSR), respiration, and more (Rosenboom, 1976, 1997, 2003a). More recently, the practice of sonification, mapping neuroscience data onto sound for artistic purposes, has been growing (ex. Minciacchi, 2011–2012). My work has emphasized using EEG features in self-organizing musical forms within feedback paradigms. The analysis methods include non-invasive techniques amenable to musical situations: spectral decomposition, coherent wave analysis, and event-related potentials (ERPs) with principal component analysis (especially N100 and P300). Recently, wearable mobile EEG technology, advances in dry electrode designs, and cost-reductions in hardware fabrication have suggested new possibilities.

**Figure 1:**
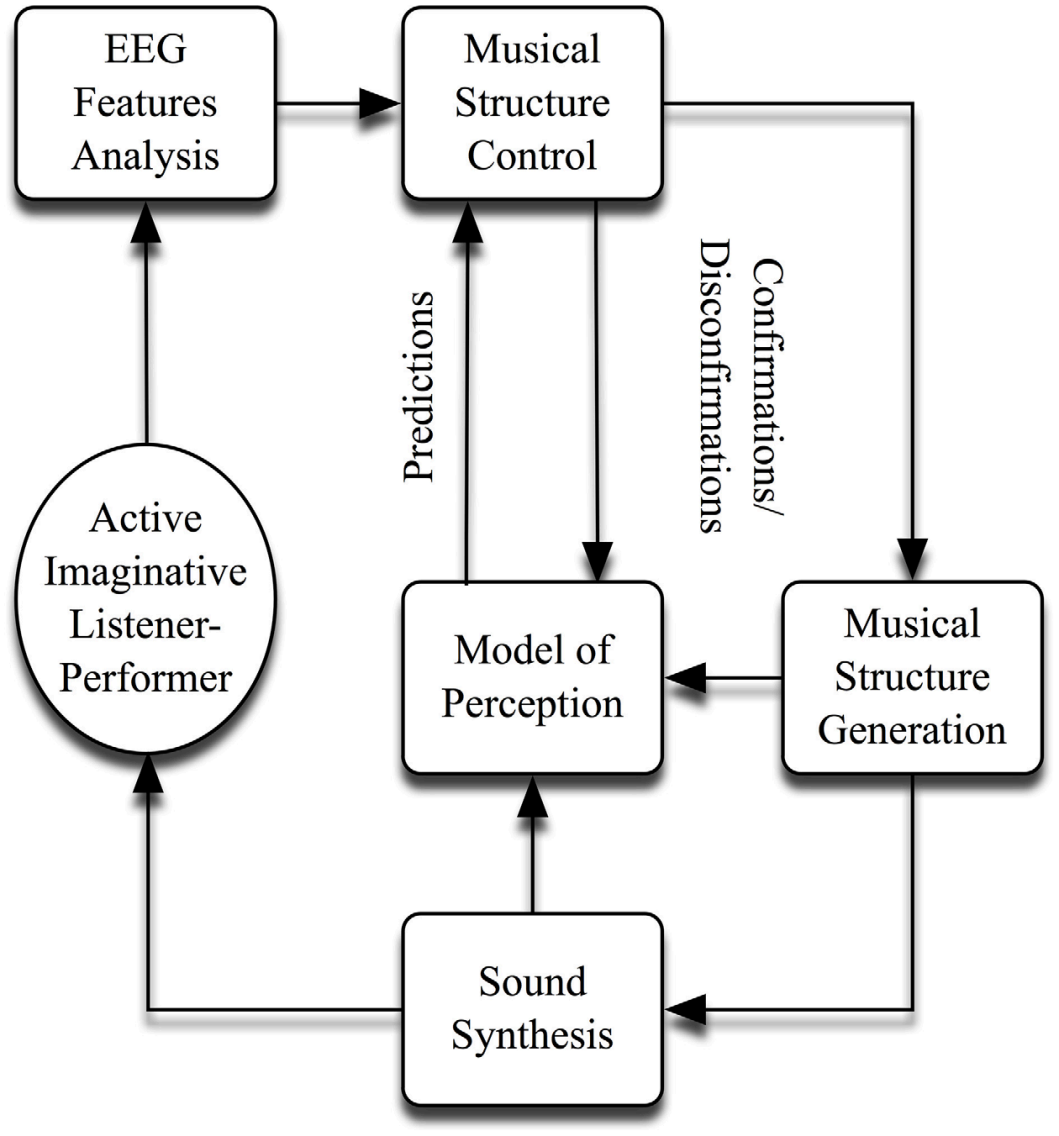
**General scheme for self-organizing neuromusic works**.

Nearly all these works are self-organizing in nature. Two of my most well-known—(originally composed in the 1970s)—are titled, *Portable Gold and Philosophers*' *Stones* and *On Being Invisible* (Rosenboom, 1997, 2000b). A generalized schematic for the implementation of these and other similar works is shown in Figure 1. All employ feedback from EEG components—(and sometimes EMG, GSR, body temperature, etc.)—recorded from *active imaginative listener-performers* in a co-evolving relationship with a system that generates and organizes electronic sound. Sometimes, extensive practicing precedes performances, in which sonic results are related to acquiring facility for enhancing or controlling particular EEG features or other phenomena. These *biofeedback* paradigms are also often used to explore subjectively identified, musical states of mind. In more involved setups, a predictive model is used to identify features in sounds produced spontaneously by composition algorithms that are likely to elicit shifts of attention in the listener-performer. These are treated as highly likely, perceptual parsing points in an emerging musical form. When the model produces predictions, confirming neural concomitants are sought, such as strong P300 waves in auditory ERPs and/or desynchronized coherent waves (alpha, beta, theta, etc.). If the predictions are confirmed in this way, the composition algorithms will evolve in a certain musical direction; and if they are disconfirmed, the music will evolve in a different way. The predictive process employs simple—certainly incomplete—models of musical perception that weigh changes in acoustic parameters (pitch, loudness, timbral complexity, noise qualities, etc.), according to their recent degrees of variance and other matters of context, with sensitivity to temporal masking effects. Associations with traditional musical styles or content are intentionally avoided, so that the system can be maximally stylistically independent. For sonic purity and simplicity, the system acts primarily on raw, acoustic features. It is also able to build musical tree structures. Once low level elements and sequences are identified by successful parsing tests, they can be stored and later recalled in hierarchically organized sequences. Another algorithm calculates expectancy values for the occurrence of individual musical elements or groups of elements in sequences, based on their temporal history, and tests for perceptual parsing when the sequences vary in particular ways. Confirming results from EEG analyses enable multiple levels of grouping to grow higher in the tree hierarchy. Disconfirming ones cause the tree to stay shallower—(see Rosenboom, 1997 for a detailed description of this process). Finally, in each performance, a unique musical form emerges, as this *attention-dependent sonic environment* self-organizes, converging upon and diverging from patterns, and patterns of patterns.

In addition to producing unique musical compositions and performances, this work suggests new ways of investigating how we might parse sonic experiences, irrespective of their association with particular styles or purported languages of music and without over-relying on presumed syntactic algorithms. Though notions of musical syntax and symbolic computation are not withstanding, these methods might help broaden our understanding of states of mind associated with diverse musical practices, particularly those found in contemporary music, experimental music, indigenous music from around the Globe, and non-Western classical music. Experiences that we may *call musical* can arise from applying *active imaginative listening* to virtually any auditory scene, sonic environment, or differentiated sonic objects. Therefore, it may be useful for both neuroscience and music to begin by collapsing distinctions among activities presumed to be musical vs. not musical, and then design useful and necessarily constrained experimental paradigms with full knowledge of their limitations. We should not succumb to a Western classical tonal myopia in music neuroscience research. The domain of creative music making drawing from work in neuroscience is expanding rapidly and moving on a path toward establishing itself in a substantial way.

## Interactivity, improvisation, neuro-composition methods, and possible next phases of creative musical neuroscience

Much music, not all, involves shared experiences and is fundamentally interactive. Ian Cross has written extensively about the many, highly-varied dimensions of music as an interactive medium for both music specialists and non-specialists in Western, non-Western, traditional, and new digital media contexts (Cross, 2013). Such interaction often involves spontaneous parsing of unpredictable musical forms, especially in improvisation. To the extent that it is communicative, i.e. involving more than one individual, it is co-creative. Masterful improvisation is one of the most demanding forms of music making. It extends spontaneous parsing to hierarchical temporal sequences. This requires maintaining increasingly large “chunks” and repertoires of *adjacent musical references* as structured improvisation—spontaneous composition—unfolds. The ability to maintain these “open frames” in working memory, as described by Fitch (2013), requires extensive practice and may require whole-brain analysis to understand. Tree structures in musical forms may be *holarchic*—(a term used to refer to structures in which organizing information flows top-down as well as bottom-up). Understanding how the brain processes the perception and apprehension of musical holarchies may require a large-scale approach to neocortical dynamic function and EEG (Nunez, 2000), along with tools for dynamical causal modeling and connectivity analysis (Marreiros et al., 2013). These also suggest exciting new possibilities for creative neuromusic.

So far, musical neuro-composition methods have evolved through these phases: (1) early observation and discovery of measurable phenomena and mapping these onto aesthetic experiences; (2) investigation of feedback and self-organizing systems with these phenomena; and (3) working with the neural concomitants for the perception of musical forms and parsing emerging sonic experiences as music. The next phases will explore *complexity* in co-adaptive neural networks, complexity in musical forms, ear training for complexity, investigating the natural ability of our auditory perception systems to *hear degrees of order*, and the complex co-creative forms of improvisation. A growing interest is emerging in music neuroscience in studying jazz improvisation, and this is a positive sign (See examples: Limb and Braun, 2008; Donnay et al., 2014). However, it should be stressed that the field of improvisation is much larger than that represented by jazz, particularly when based on traditional jazz forms, and a host of alternatives offers rich opportunities for further study—(see Bailey, 1992 for an example of a broad approach to improvisation).

Key to this will be research in understanding how we process complexity. Music is ideal for this study. Holistic imaging of brain activity in real-time and during complex musical interactivity will be essential for pushing this agenda further. Preliminary results already indicate that *dimensional complexity analyses* of music stimuli and EEG activity may be closely related to each other and affected by musical experience (Birbaumer et al., 1996). This suggests ways to extend my earlier work exploring self-organizing musical forms guided by feedback from auditory ERPs (P300) and correlating model predictions with confirmations or disconfirmations of attention shifts to key features of change in musical forms. Affective studies on aesthetics from decades ago already investigated perception of and preferences for amounts and types of variance and complexity in musical sequences—(early studies are summarized in Berlyne, 1971). We now talk of *complexodynamics* in musical composition, in which we work with relationships among entropy, complexity, and interestingness. We know from musical experiences that we can develop keen sensitivity to and incisive parsing and comparison skills for subtle changes in the complexity of auditory scenes. For instance, we can track how people learn to hear differences and make comparisons among stochastic clouds of sound and among natural and artificial soundscapes. This has resulted in new kinds of music learning and ear training, including pedagogical methods for hearing sonic forms, in which the primary organizing principles are not the traditional ones of melody, harmony, and rhythm, called *spectromorphology* (Trayle, 2014). It could be fruitful for both musical artistry and music neuroscience to explore the possibilities of musical forms that might emerge from applying complexity analysis to the self-organizing feedback paradigms of earlier work.

These projects might extend possibilities for interactive, intelligent musical instruments as well, in which relationships among the complex networks of performing brains and adaptive, algorithmic musical instruments can become *musical states*, ordered in compositions like notes and phrases (Rosenboom, 1992). New ways of extending this with human–computer interface (HCI) may be upon us (ex. Miranda and Wanderley, 2006). New practices for brain awareness and self-organizing musical forms may also result. A new project in group-brain, musical performance, undertaken by the author with colleagues at the Schwartz Center for Computational Neuroscience, Institute for Neural Computation, University of California San Diego (UCSD), made use of techniques developed originally for epilepsy research (Mullen et al., 2012). Principal oscillation patterns (POPs or *eigenmodes*) were extracted from the EEGs of five individuals, along with auditory ERPs averaged across the five brains—(instead of across time)—, treating the data as if it arose from a five-person collective brain. A computer sound synthesis instrument, the core of which consists of a large network of complex resonators, was programmed to enable mapping data from the EEG eigenmodes, onto an expansive, spatialized sound field produced with the resonators. The performance also involved two live performers who interacted with the sound field carefully, so as to potentially influence the ERPs, which would in turn modulate the sound field (Rosenboom et al., 2014).

Music neuroscience might benefit from closer integration with advanced studies in musical modeling that have been growing for a long time. Even for such an obvious musical parameter as pitch, the surface has only been scratched. We may assume the brain has evolved efficient processing mechanisms for pitch and timbre, and these are still being uncovered. Mathematical studies in efficient pattern recognition algorithms for modeling pitch spaces may be able to guide neural network investigations further—(a striking example is found in Rothenberg, 1978a,b,c). Treating musical entities as shapes or contours with degrees of curvature, finding neural concomitants for similarity measures among a wide range of complex sonic entities, context sensitive parsing—(in my opinion, context free parsing theories offer little of relevance to understanding the nearly always, context sensitive aspects of musical forms)—, neural concomitants for imagined musical events (endogenous factors), and exploring multi-dimensional musical *concept spaces* are all areas for potentially rich investigations in music neuroscience. In the end, *active imaginative listening* to the musical potential in all sound may offer a simple beginning to which a periodic return may be helpful.

### Conflict of interest statement

The author declares that the research was conducted in the absence of any commercial or financial relationships that could be construed as a potential conflict of interest.

## Links

Link to the author's work as composer-performer, interdisciplinary artist, author, and educator:

Main Website: http://www.davidrosenboom.com/

Links to selected recordings containing compositions by the author:

*Zones of Influence*: http://www.pogus.com/21074.html

*Life Field*: http://www.tzadik.com

*How Much Better if Plymouth Rock Had Landed on the Pilgrims*: http://www.newworldrecords.org/album.cgi?rm=view&album_id=82691

*In the Beginning*: http://www.newworldrecords.org/album.cgi?rm=view&album_id=91267

*Future Travel*: http://www.newworldrecords.org/album.cgi?rm=view&album_id=81485

*Invisible Gold*: http://www.pogus.com/21022.html

*Two Lines*: http://www.lovely.com/titles/cd3071.html

*Roundup Two*: http://www.art-into-life.com/product/2251

*Suitable for Framing*: http://mutablemusic.com/mm/framinginfo
